# Pruritus Vulvæ, Treatment

**Published:** 1874-12

**Authors:** 


Treatment of Pruritus Vulvæ.—*La France Medicale* gives the following recipes:

Alum lotions may be prescribed according to the following formula:

℞.—Alum, . . . . . . . . . . ʒj

Barley water, . . . . . . . . ℥xvj. M.

For a lotion, to be used three or four times a day, M. Harding frequently employed the following:

℞.—Corrosive Sublimate, . . . . . gr. xv.
Distilled Water, . . . . . . . ℥iij
Alcohol, . . . . . . . . . q. s.

M.—A spoonful in a glass of tepid water. Care should be taken to avoid rubbing the parts when using this lotion.

In the pruritus which so often accompanies pregnancy, Danyau employs the following:

℞.—Oxide of Zinc, . . . . . . . ℥j

Borax, . . . . . . . . . ʒss

Simple Cerate, . . . . . . . . ℥ss

Almond Oil, . . . . . . . . q. s.

Hydrochlorate of Morphia, . . . . gr. iijss M.

M. Bazin prescribes this liniment:

℞.—Lime Water, . . . . . . .

Glycerine, . . . . . . aa ℥j

Almond Oil, . . . . . . . ℥ij. M.

He also advises the following formula:

℞.—Glycerine, . . . . . . . . . ℥v

Laudanum, . . . . . . . . ʒj

Essence of Roses, . . . . . . . gtt. v. M.

